# Epidemiology, morphology, and molecular characterization of *Stephanurus dentatus* (Nematoda: Syngamidae) in wild boars from southwestern South Korea

**DOI:** 10.1186/s13071-026-07303-6

**Published:** 2026-04-10

**Authors:** Kyu-Sung Ahn, Ah-Jin Ahn, Seung-Hun Lee, Dongmi Kwak, Jina Lee, SungShik Shin

**Affiliations:** 1 BIOREEDS Inc., 2-209 College of Veterinary Medicine, Chonnam National University, Gwangju, 61186 Republic of Korea; 2 Laboratory of Veterinary Parasitology, College of Veterinary Medicine, Chonnam National University, Gwangju, 61186 Republic of Korea; 3 Health and Environment Research Institute of Gwangju, Gwangju, 61027 Republic of Korea; 4 College of Veterinary Medicine, Chungbuk National University, Cheongju, 28644 Republic of Korea; 5 College of Veterinary Medicine, Kyungpook National University, Daegu, 41566 Republic of Korea; 6 Laboratory of Veterinary Virology, College of Veterinary Medicine, Chonnam National University, Gwangju, 61186 Republic of Korea

**Keywords:** *Stephanurus dentatus*, Wild boars, Republic of Korea, Epidemiology, Phylogenetic analysis

## Abstract

**Background:**

*Stephanurus dentatus* Diesing, 1839 (Nematoda: Syngamidae), commonly known as the swine kidney worm, parasitizes the renal pelvis, ureters, and perirenal fat of pigs and wild boars, occasionally causing severe pathological effects. Despite reports of low prevalence in domestic pigs in South Korea, no epidemiological data exist for wild boars. This study aimed to investigate the prevalence, morphology, and molecular characteristics of *S. dentatus* infection in wild boars from the southwestern regions of South Korea.

**Methods:**

A total of 167 wild boars were examined between 2009 and 2019. Kidneys, ureters, and perirenal fat were dissected, and worms were collected for morphological analysis using light and scanning electron microscopy. Molecular identification was conducted via polymerase chain reaction (PCR) and sequencing of the 18S rRNA gene. Phylogenetic analyses were performed to assess taxonomic placement. Morphological identification keys were provided for both the traditional family Syngamidae and the revised family Chabertiidae.

**Results:**

*Stephanurus dentatus* was detected in 38.3% of the examined wild boars (64/167), with a mean intensity of 6.8 worms per infected animal. Morphological analyses of adult worms revealed characteristics consistent with previous descriptions, including the corona radiata, the vulva located posteriorly in females, and a rudimentary copulatory bursa in males. Molecular analyses confirmed 99.7–99.9% identity with reference sequences and supported placement of *S. dentatus* within the family Chabertiidae. Revised identification keys based on morphology were provided.

**Conclusions:**

This study provides the first epidemiological data on *S. dentatus* in wild boars in South Korea, demonstrating a substantial prevalence and potential risk for transmission to domestic pigs. Detailed morphological descriptions, identification keys, and molecular analyses presented here contribute valuable information for faunistic, taxonomic, and parasitological studies of *S. dentatus*.

**Graphical abstract:**

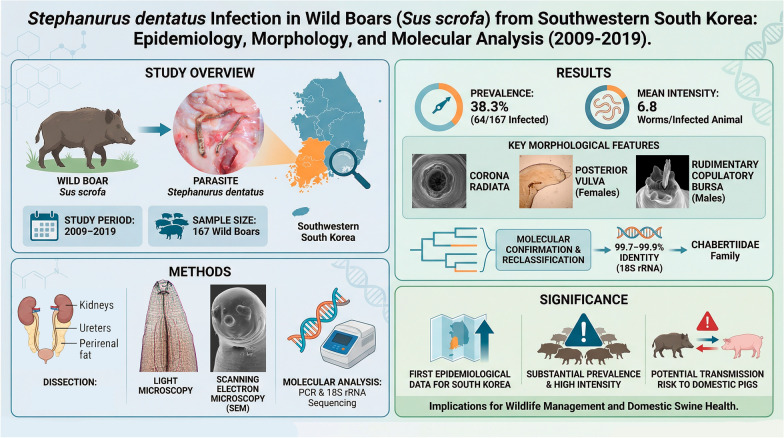

**Supplementary Information:**

The online version contains supplementary material available at 10.1186/s13071-026-07303-6.

## Background

*Stephanurus dentatus* Diesing, 1839 (Nematoda: Syngamidae), also known as the swine kidney worm, parasitizes the renal pelvis, perirenal fat, and ureteral walls of pigs [1, 2]. Occasionally, they are found ectopically in organs such as the liver, thoracic organs, abdominal organs, and spinal canal. The fourth stage larvae (L4) can inflict extensive damage to the liver and, less frequently, other organs during migration to their final host location. Heavy infections can result in severe cirrhosis, thrombosis of hepatic vessels, and ascites [3]. Infection occurs via skin penetration, ingestion of infective third-stage larvae (L3) in earthworms acting as transport hosts, or ingestion of L3 from feces-contaminated food sources or water [3]. Rare infections in other species, including cattle and donkeys, have also been documented [4].

*Stephanurus dentatus* is prevalent in tropical and subtropical regions worldwide, with particularly high infection rates among pigs and wild boars in Spain (76.5%) [5], Brazil (71.4%) [6], Papua New Guinea (65.2%) [7], Japan (55.2%) [8], Nepal (44.0%) [9], Belize (42.0%) [10], India (40.5%) [11], and Ghana (33.0%) [12]. In contrast, low infection rates have been reported in the USA (3.1%) [13], Nigeria (1.1–4.9%) [14, 15], and Ghana (1.8%) [16].

In Korea, previous studies reported a low prevalence of *S. dentatus* in domestic pigs: 0.5% in 1962 (Lee, 2550 pigs) [17] and 1.3% in 1994 (Yang, 662 pigs) [18]. In wild boars, only a single case has been reported (Suh, 2002) [19], and no studies have yet addressed the prevalence of this parasite among wild boars in Korea. In the present study, we examined wild boars from the southwestern region of the Republic of Korea (South Korea) between 2009 and 2019 and found a high prevalence of *S. dentatus* infection.

Furthermore, we generated molecular sequence data and provide a detailed morphological description, including morphometric measurements of key structures in adult males and females, together with identification keys. Although *S. dentatus* has traditionally been classified in the family Syngamidae (superfamily Strongyloidea [20], recent phylogenetic studies using mitochondrial genomes and nuclear rDNA markers suggest a closer affinity with Chabertiidae and propose that *S. dentatus* may be more appropriately placed in that family [21, 22]. Accordingly, we conducted phylogenetic analyses on the basis of the 18S rRNA gene to further evaluate its taxonomic placement. In addition, we present two alternative identification keys for *S. dentatus*, reflecting its placement under either Syngamidae or Chabertiidae.

## Methods

### Animals

A total of 167 wild boars (*Sus scrofa coreanus*) hunted in the mountains of Suncheon-si (si = city, 10 males, 18 females), Gwangyang-si (80 males, 53 females), Boseong-gun (gun = county, 4 females), and Haman-gun (1 male, 1 female) were donated by local hunting associations over an 11-year period (2008–2019 Additional file 1: Supplementary Fig. S1). The Korean government supports wild boar population control via licensed hunters, targeting areas with significant crop damage or disease prevalence.

Organs of each animal were removed by hunters on-site within 1 h after shooting, and the urogenital organs were brought to the Laboratory of Parasitology at Chonnam National University College of Veterinary Medicine individually in ice box immediately after processing. The kidneys, ureters, and the perirenal fat and tissues were carefully dissected and were examined macroscopically for helminth parasites. Collected worms were stored in 70% ethanol at room temperature for morphological study and 99% ethanol at −20 °C for molecular analysis.

### Light microscopy

Worms collected from infected animals preserved in 70% ethanol were mounted on slides using the polyvinyl alcohol mounting medium [23]. Measurements of adult male and female body parts were obtained with a light microscope (Axioskop, Zeiss, Germany). Species identification followed Anderson et al. [24].

### Scanning electron microscopy

Worms were washed in phosphate-buffered saline three times for 20 min each, fixed in 2.5% glutaraldehyde in 0.1 M phosphate buffer (pH 7.2) for 24 h at 4 °C, and post-fixed in 1% OsO_4_ for 2 h at 4 °C. After serial dehydration in 30%, 50%, 70%, 80%, 90%, and 100% ethanol for 20 min each, worms were treated with critical-point drying in a Hitachi HCP-2 (Hitachi Ltd., Tokyo, Japan) and sputter-coated with gold–palladium in an Emitech K550 (Emitech Ltd., Ashford, Kent, England). Digital photographs of worms were taken using a Hitachi S-2400 scanning electron microscope (Hitachi Ltd., Tokyo, Japan).

### Morphological identification keys

Diagnostic features were illustrated in Adobe Illustrator CS6. Identification keys were adapted from Lichtenfels [20] and Daubney [25], and were presented under both Syngamidae and Chabertiidae to reflect alternative familial placements proposed in recent molecular studies [21, 22].

## DNA extraction and polymerase chain reaction

Molecular identification was performed on four female adult worms (SD 1–SD 4) isolated from different host animals hunted in Suncheon-si, Gwangyang-si, and Boseong-gun. Selected worms were washed three times with sterile phosphate-buffered saline. DNA extraction was conducted using the QIAamp^®^ DNA Mini Kit (QIAGEN, Hilden, Germany) according to the manufacturer’s instruction.

Polymerase chain reaction (PCR) was performed using Solg™ 2X Multiplex PCR Smart mix (Solent, Daejeon, Korea). The 18S rRNA gene of *S. dentatus* was amplified using the primer set NC18SF1 (5′-AAA GAT TAA GCC ATG CA-3′) and NC5BR (5′-GCA GGT TCA CCT ACA GAT-3′) [26]. The PCR thermal cycling conditions consisted of an initial denaturation at 95 °C for 15 min, followed by 40 cycles of denaturation at 95 °C for 30 s, annealing at 60 °C for 30 s, and extension at 72 °C for 1 min, with a final extension step at 72 °C for 5 min.

### Sequencing and phylogenetic analysis

DNA sequencing was performed using the BigDye^®^ Terminator v3.1 Cycle Sequencing Kit (Applied Biosystems, Waltham, MA, USA) and an ABI 3730xl DNA Analyzer (Applied Biosystems). Of the nucleotide sequences obtained, low-quality sequences (~100 base pairs (bp) from each end) were trimmed prior to analysis using BioEdit v.7.2.5 and MEGA v.11.0 software. A phylogenetic tree was constructed using the maximum likelihood method with 1000 bootstrap replicates.

### Statistical analysis

Infection prevalence between sexes was compared using Fisher’s exact test, and odds ratios with 95% confidence intervals (CIs) were calculated to assess the strength of association. To assess sex-associated differences in *S. dentatus* worm burden, analyses were performed on infected animals only (total worm count > 0). Worm burden was quantified as the number of male worms, female worms, and total worms recovered per host. Differences in worm burden between male and female hosts were evaluated using Mann–Whitney *U* tests (two-sided). In addition, negative binomial regression was used to model count outcomes and account for overdispersion, with results reported as incidence rate ratios (IRRs) with 95% CIs. Statistical significance was set at *P* < 0.05. Within infected hosts, paired numbers of female and male worms were compared using two-sided Wilcoxon signed-rank tests, stratified by host sex. In addition, worm sex composition was analyzed using a binomial generalized linear model (GLM) (logit link) with the number of female worms out of the total sexed worms per host as the response and host sex as the predictor; results are presented as odds ratios (ORs) with 95% CIs. Statistical analyses were performed in R.

## Results

### Prevalence and worm burden

Out of 167 wild boars captured in the southwestern regions of South Korea, 64 (38.3%) were found to harbor kidney worms in the renal pelvis, the perirenal fat, and ureteral walls. Worms were found in 5 out of 28 wild boars (17.9%) from Suncheon-si, 56 out of 133 (42.1%) from Gwangyang-si, and 3 out of 4 (75.0%) from Boseong-gun. None of the two animals from Haman-gun were infected with kidney worms (Table 1).

**Table 1:** Prevalence and worm burdens of *Stephanurus dentatus* in wild boars by sex and location in southwestern South Korea

Area	Sex	No. of animals			Infection rate (%)	No. of adult worms			Mean no. per animal^a^	F/M ratio^b^
		Infected	Uninfected	Total		Female	Male	Total		
Suncheon-si	Male	2	8	10	20.0	7	6	13	6.5	1.2
	Female	3	15	18	16.7	10	9	19	6.3	1.1
	Total	5	23	28	17.9	17	15	32	6.4	1.1
Gwangyang-si	Male	35	45	80	43.8	137	110	247	7.1	1.2
	Female	21	32	53	39.6	88	60	148	7.0	1.5
	Total	56	77	133	42.1	225	170	395	7.1	1.3
Boseong-gun	Male	0	0	0	0.0	0	0	0	0	0.0
	Female	3	1	4	75.0	6	5	11	3.7	1.2
	Total	3	1	4	75.0	6	5	11	3.7	1.2
Haman-gun	Male	0	1	1	0.0	0	0	0	0	0.0
	Female	0	1	1	0.0	0	0	0	0	0.0
	Total	0	2	2	0.0	0	0	0	0	0.0
Total	Male	37	54	91	40.7	144	116	260	7.0	1.2
	Female	27	49	76	35.5	104	74	178	6.6	1.4
	Total	64	103	167	38.3	248	190	438	6.8	1.3

^a^Mean number of adult worms per animal

^b^Female-to-male ratio of adult worms (F:M)

Prevalence was 40.7% in male wild boars (37/91) and 35.5% in female wild boars (27/76), with no significant difference between sexes (Fisher’s exact test, *P* = 0.48; OR = 1.25, 95% CI 0.67–2.34). A total of 438 worms (248 females and 190 males) were collected, with an average of 6.8 worms per infected animal. Mean total worm burden among infected boars was very similar between sexes (7.0 in males versus 6.6 in females), and nonparametric comparisons (Mann–Whitney *U* tests) showed no significant differences in male worm burden (*P* = 0.651), female worm burden (*P* = 0.418), or total worm burden (*P* = 0.492).

Among 64 infected wild boars, female worms were more numerous than male worms in both host sexes (male hosts: 144 female versus 116 male worms; female hosts: 104 female versus 74 male worms). The female-to-male worm ratio was 1.24 in male hosts and 1.41 in female hosts (overall F:M = 1.31, 248/190). In within-host paired analyses, female wild boars harbored significantly more female worms than male worms (Wilcoxon signed-rank test, *P* = 0.008), whereas the difference was not statistically significant in male wild boars (*P* = 0.086). In a binomial GLM evaluating worm sex composition, the proportion of female worms did not differ between female and male wild boars (female versus male: OR = 1.07, 95% CI 0.75–1.53, *P* = 0.70), indicating no evidence that host sex influenced the sex ratio of recovered worms.

### Morphology

**Fig. 1:**
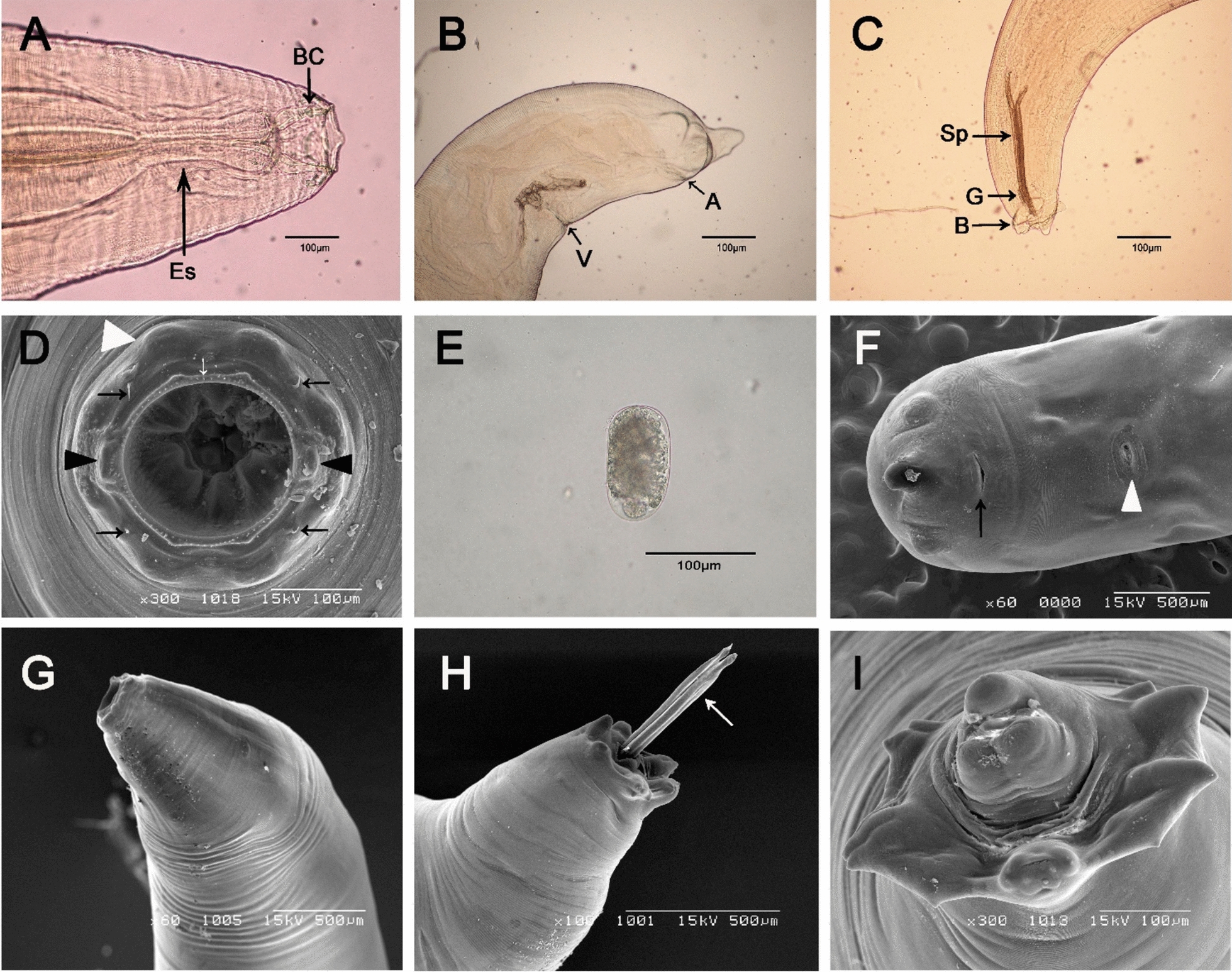
Light and scanning electron micrographs of *Stephanurus dentatus* recovered from the kidneys and perirenal adipose tissue of Korean wild boars (*Sus scrofa coreanus*) in South Korea. **A** Lateral view of the anterior end of a female, showing the buccal capsule (BC) and esophagus (Es). **B** Lateral view of the posterior end of a female, showing the vulva (V) and anus (A). **C** Lateral view of the posterior end of a male, showing the spicules (Sp) and copulatory bursa (B). **D** Enlarged en face view of the anterior end, showing two lateral cephalic papillae (black arrowhead), four submedian cephalic papillae (black arrows), and the corona radiata (white arrow). **E** Egg of *S. dentatus*. **F** Posterior end of a female, showing the anus (black arrow) and vulva (white arrowhead). **G** Enlarged view of the anterior end, showing the cephalic region. **H** Enlarged view of the posterior end of a male, showing two spicules (white arrows). **I** Enlarged view of the posterior end of a male, showing the copulatory bursa. **D**, **F–I** are scanning electron micrographs; others are light micrographs

The average length of adult males was 26.1 ± 4.3 mm, with the thickest part of the body measuring 1.1 ± 0.1 mm, while females averaged 35.8 ± 4.3 mm in length and 1.8 ± 0.5 mm in thickness (Tables 2, 3). The average length of the buccal capsule of males and females was 181.0 µm and 210.5 µm, respectively (Fig. 1A, D). The average length and width of ellipsoidal eggs excreted from the ovijector of females were 110.3 µm and 66.5 µm, respectively (Fig. 1E). The average length of the identical pair of spicules of males was 1006.0 ± 91.6 µm (Tables 2, 3).

**Table 2:** Morphometric measurements of adult male *Stephanurus dentatus* from wild boars in South Korea compared with previous reports

Sex	Anatomical location	Measurements of body parts (µm)				
		Present study	Suh (2002)	Daubney (1923)	Skrjabin (1921)	Tayler (1900)
Male	Number of worms	10	16	–	–	–
	Body length	26,087.2.0 ± 4272.9 ^a^	25,100 ± 3200	–	–	–
		(21,500.0–33,150.0) ^b^	–	(20,000–28,000)	–	–
	Body width	1061.0 ± 131.9	–	–	–	–
		(840.0–1320.0)	–	(~1200)	–	–
	Length of buccal capsule	181.0 ± 34.9	–	180	–	–
		(135.0–260.0)	–	–	–	–
	Width of buccal capsule	205.0 ± 34.9	–	–	–	–
		(120.0–255.0)	–	–	–	–
	Distance of excretory pore from anterior end	983.0 ± 37.8	–	–	–	–
		(1050.0–940.0)	–	–	–	–
	Length of esophagus	1271.0 ± 345.8	–	–	–	–
		(1120.0–1420.0)	–	–	–	–
	Length of dorsal ray	118.0 ± 28.3	–	–	–	–
		(80.0–170.0)	–	–	–	–
	Length of externodorsal ray	101.5 ± 20.1	–	–	–	–
		(75.0–145.0)	–	–	–	–
	Length of spicules	1006.0 ± 91.6	–	800.0	935.0	800.0
		(860.0–1170.0)	–	–	–	–

^a^Average ± standard deviation

^b^Range

**Table 3:** Morphometric measurements (μm) of adult female *Stephanurus dentatus* (and eggs) from wild boars in South Korea compared with previous reports

Sex	Anatomical location	Measurements of body parts (µm)		
		Present study	Suh (2002)	Daubney (1923)
Female	Number of worms	10	11	–
	Body length	35,774.0 ± 4272.0^a^	34,200.0 ± 2900.0	25,000.0–45,000.0
		(28,720.0–42,500.0) ^b^	–	–
	Body width	1775.0 ± 504.9	–	–
		(1020.0–2500.0)	–	(~1800.0)
	Length of buccal capsule	210.5 ± 49.5	–	1800.0
		(120.0–275.0)	–	–
	Width of buccal capsule	236.0 ± 55.9	–	–
		(120.0–300.0)	–	–
	Distance of excretory pore from anterior end	1128 ± 51.8	–	–
		(1200.0–1050.0)	–	(500.0–600.0)
	Length of esophagus	1356.0 ± 211.4	–	1600
		(820.0–1580.0)	–	–
	Distance of vulva from anus	1106.0 ± 209.0	–	1360
		(750.0–1350.0)	–	–
	Distance of anus from posterior end	481.0 ± 119.4	–	–
		(250.0–620.0)	–	–
	Length of eggs (*n* = 10)	110.3 ± 3.1	–	–
		(105.0–115.0)	(90.0–115.0)	(90.0–100.0)
	Width of eggs (*n* = 10)	66.5 ± 4.0	–	–
		(57.0–70.0)	(40.0–65.0)	(56.0–65.0)

^a^Average ± standard deviation

^b^Range

**Fig. 2:**
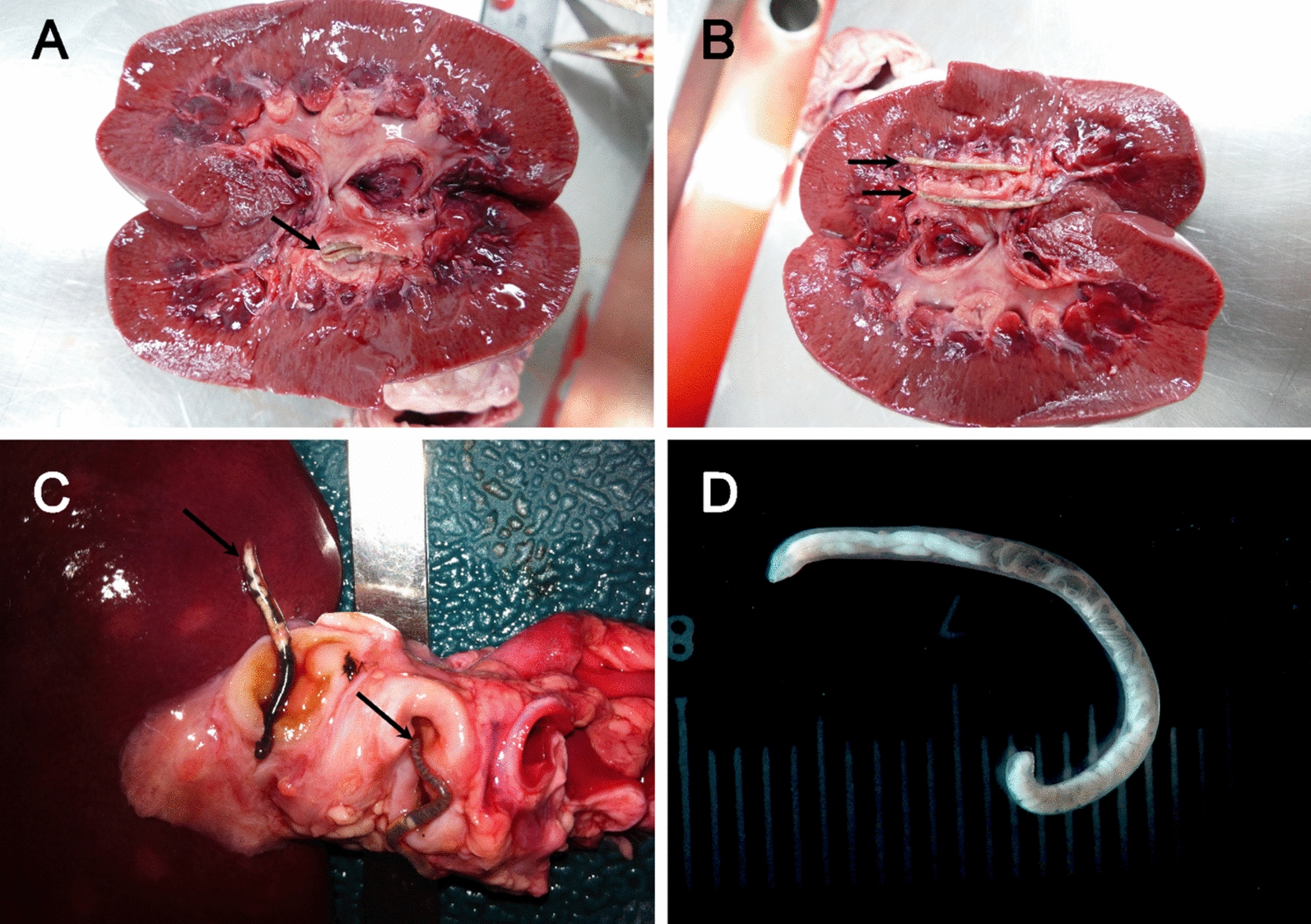
Stereomicroscopic images of *Stephanurus dentatus* collected from wild boar kidneys and perirenal fat at necropsy. **A, B** Encysted adult worms before and after removal from the renal pelvis. **C** Adult worms enclosed within cysts in the perirenal fat. **D** Adult female isolated from the kidney; note the transparent cuticle

The mouth of the worm was circular, with a corona radiata of small, pointed elements around the mouth opening and a relatively thick-walled, cup-shaped buccal capsule inside. There were two median and four sub-median papillae surrounding the mouth, and four thorn-shaped sub-median papillae between the median and sub-median papillae (Fig. 1A, D, G). The posterior extremity of female worms tapered sharply toward the end. The vulva and anus were located at the posterior end of the female and were adjacent. The vulva had an elongated, circular opening, while the anus appeared as an elongated, semicircular hole (Fig. 1B, F). The male copulatory bursa was small and short, and the equal pair of spicules were either protruding outside or hidden inside (Fig. 1C, H, I). Adult worms (Fig. 2D) were found in the renal pelvis of the kidney (Fig. 2A, B), along the ureters in the perirenal fat (Fig. 2C), and in the walls of the ureter.

### Molecular analysis

**Fig. 3:**
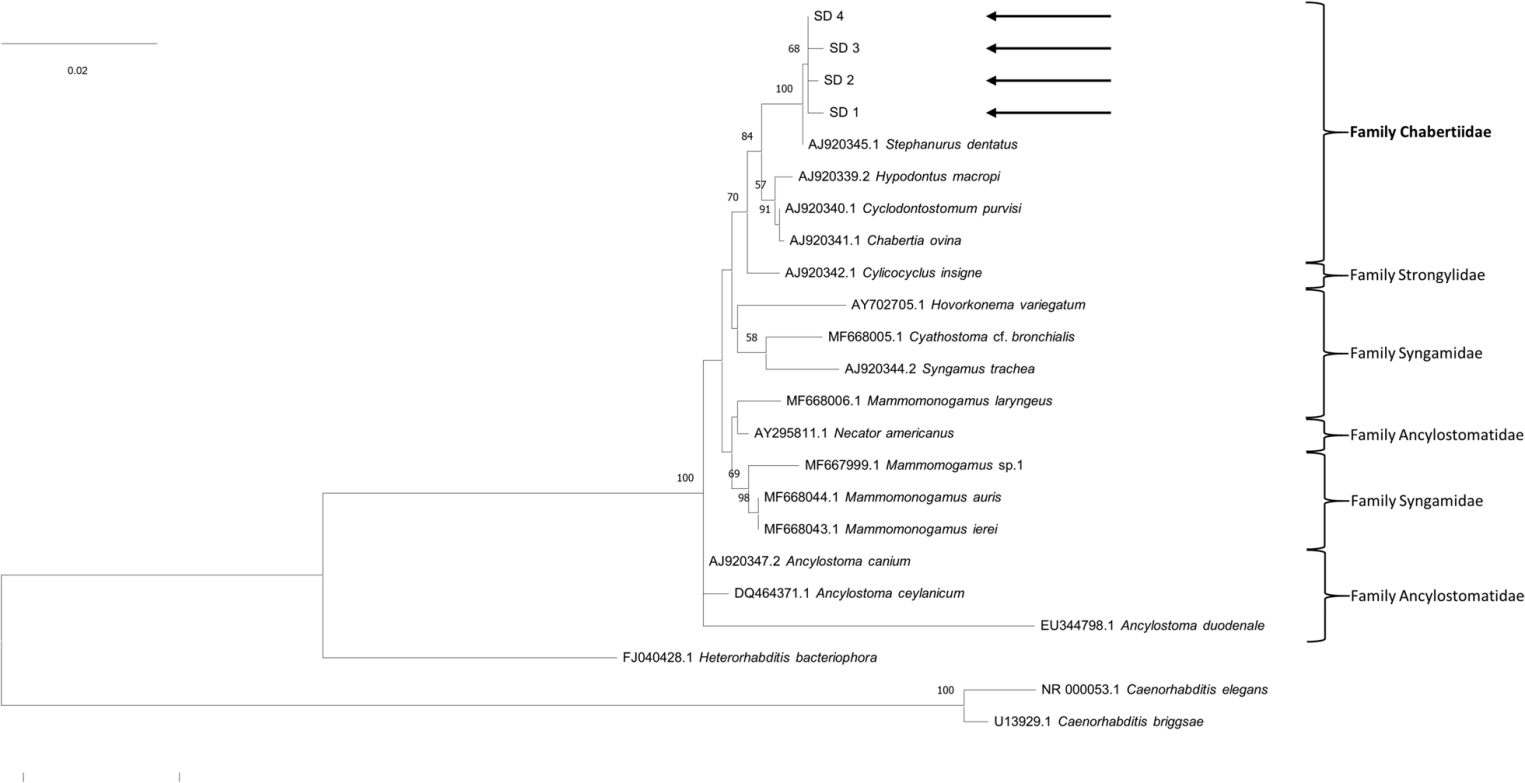
Maximum-likelihood phylogenetic tree of *Stephanurus dentatus* based on 18S rRNA gene sequences. Bootstrap support values were calculated from 1000 replicates. Sequences generated in the present study are indicated by arrows (SD1–SD4)

Sequence analysis and Basic Local Alignment Search Tool revealed that the four *S. dentatus* 18S rRNA genes in this study showed 99.7–99.9% identity with the *S. dentatus* (AJ920345) in the GenBank database. In addition, phylogenetic analysis showed that the four 18S rRNA genes of *S. dentatus* closely belonged to the family Chabertiidae than to the family Syngamidae (Fig. 3). The sequences obtained in this study were submitted to the GenBank database (accession nos. PX762437–PX762440).

## Discussion

### Prevalence and spatial heterogeneity

Approximately 70% of the land area of the Republic of Korea is mountainous, and the absence of apex predators such as tigers, leopards, and wolves has likely contributed to increases in wildlife populations that were previously regulated by predation. Consequently, numerous wild mammals—including wild boars, raccoon dogs, badgers, water deer, roe deer, and hares—now inhabit both mountainous and agricultural landscapes. Among these species, the wild boar (*Sus scrofa coreanus*) has been identified as a major source of crop damage in Korea [27–29].

In addition to the economic losses described above, wild boars may act as reservoirs and/or vectors of infectious diseases. In Korea, serologic surveys of wild boars have detected antibodies to porcine circovirus type 2 (PCV2), porcine reproductive and respiratory syndrome virus (PRRSV), classical swine fever virus (CSFV), Japanese encephalitis virus (JEV), and foot-and-mouth disease virus (FMDV) [30–33]. However, the prevalence of *Stephanurus dentatus* in Korean wild boars has not yet been systematically investigated.

In this study, *Stephanurus dentatus* infection was common in wild boars from southwestern South Korea, with an overall prevalence of 38.3% (64/167). Adult worms were recovered from the renal pelvis and were also detected in perirenal fat and the ureteral wall, which is consistent with the species’ predilection for tissues surrounding the urinary system. This distribution highlights the need to examine perirenal tissues and ureters in addition to opening the renal pelvis. Restricting inspection to the kidney lumen alone may lead to underestimation of infection and worm burden.

Prevalence varied across sampling areas, being 17.9% in Suncheon-si, 42.1% in Gwangyang-si, and 75.0% in Boseong-gun, and no infection was detected in the two animals examined from Haman-gun. These findings suggest spatial heterogeneity and potentially focal transmission. However, the site sample sizes were strongly unbalanced, and the estimates for Boseong-gun (*n* = 4) and Haman-gun (*n* = 2) should be interpreted with caution. The high prevalence observed in Gwangyang-si, which contributed the largest number of animals, supports established local transmission and suggests that ecological or landscape-level factors may influence exposure in this region.

### Host sex effects on infection and worm burden

Host sex was not associated with infection status. Prevalence was 40.7% in males (37/91) and 35.5% in females (27/76), and the difference was not significant (Fisher’s exact test, *P* = 0.48; OR = 1.25, 95% CI 0.67–2.34). Worm burden among infected animals was also similar between sexes, with a mean intensity of 7.0 worms in males and 6.6 worms in females. Mann–Whitney *U* tests showed no significant differences in male worm burden (*P* = 0.651), female worm burden (*P* = 0.418), or total worm burden (*P* = 0.492). Together, these results suggest comparable exposure and/or susceptibility in male and female wild boars in the study area, and they indicate that geographical factors may be more informative than host sex for identifying higher-risk groups.

### Worm sex ratio patterns and interpretation

Across infected hosts, worm populations were female biased, and female worms outnumbered male worms in both host sexes (male hosts F:M = 1.24; female hosts F:M = 1.41). Although the female bias was numerically higher in female hosts, regression-based analysis of worm sex composition did not indicate that host sex affected the probability that a recovered worm was female (binomial GLM, OR = 1.07, 95% CI 0.75–1.53). This pattern is therefore more consistent with parasite-level processes than with a strong host sex effect. Possible explanations include differential survival or longevity of female worms, sex-specific establishment success, or differences in recovery or detectability. Overall, the lack of host sex differences in prevalence and intensity supports the view that local ecological conditions are more important than host sex in shaping infection patterns in this system, while the observed female-biased sex ratio likely reflects intrinsic features of *S. dentatus* population biology within hosts.

### Epidemiology in Korea

This study indicates that wild boars from the southwestern mountainous regions of South Korea were heavily infected with *Stephanurus dentatus*, with an overall prevalence of 38.3%. This prevalence is considerably higher than previous reports in domestic pigs (0.5% in 1962 [17] and 1.3% in 1994 [18]) and represents the first epidemiological assessment of *S. dentatus* infection among wild boars in Korea.

The lower prevalence in domestic pigs likely reflects the widespread use of anthelmintics and reduced exposure to earthworms in modern pig farming systems [34]. However, the detection of *S. dentatus* eggs by fecal examination may also reflect methodological bias, as both previous Korean studies relied solely on fecal examination rather than urine analysis or the identification of adult worms in or around the kidneys. Because *S. dentatus* eggs are excreted primarily in urine rather than feces, their detection in fecal samples is likely due to urine contamination, particularly when samples are collected from pen floors. In natural or appropriately designed housing environments, pigs typically urinate and defecate in the same designated area, which is deliberately located away from their lying and feeding areas [35].

### Global epidemiology

The prevalence observed in Korean wild boars (38.3%) is lower than in some tropical and subtropical regions, such as Japan (55.2% [8]), Nouvelle-Calédonie (64.3% [36]), Spain (78% [5]), and Brazil (71.4% [6]). Across the available necropsy-based surveys (including the present study), the crude pooled prevalence in wild boars was 56.6% (228/403; Table 4). In contrast, the corresponding crude pooled prevalence in domestic pigs was 33.6% (2688/7995) across nine necropsy-based studies (Table 4). When these aggregated animal-level counts are compared, wild boars show a higher probability of infection than domestic pigs (RR = 1.68, 95% CI 1.52–1.86; *P* < 0.001), although this estimate should be interpreted cautiously because it is based on crude pooling and does not account for between-study heterogeneity (e.g., geography, study period, production system, and diagnostic intensity) that would ideally be handled in a random-effects meta-analytic framework.

**Table 4:** Worldwide infection status of *Stephanurus dentatus* in domestic pigs and wild boars

Method	Animals	Year surveyed	Country	No. examined	No. positive	Prevalence (%)	First author
*Adult worms by necropsy or abattoir survey*							
		1964–1967	Ghana	5869	1937	33.0	Sapong [12]
		1966–1969	Papua New Guinea	92	60	65.2	Talbot [7]
		1968–1969	USA	96	3	3.1	Riddle [13]
		Not available	Cuba	400	140	35.0	Rodriguez [43]
	Domestic pigs	1985–1989	Belize	125	53	42.4	Gibbens [10]
		1990–1992	India	1154	467	40.5	Singh [11]
		2005–2006	Bangladesh	30	3	10.0	Islam [44]
		2012–2013	St Kitts	59	10	16.9	Morosco [45]
		2020–2021	India	170	15	8.8	Padmini [46]
	Subtotal			7995	2688	33.6	
*Adult worms by necropsy*							
		2005–2006	Japan	29	16	55.2	Sato [8]
		2008–2019	Republic of Korea	167	64	38.3	Present study
	Wild boars	2012–2013	New Caledonia	70	45	64.3	Cauquil [36]
		2016–2017	Spain	102	78	76.5	Moratal [5]
		2016–2017	Brazil	35	25	71.4	Perin [6]
	Subtotal			403	228	56.6	
*Fecal egg counts*							
		1962	Republic of Korea	2550	13	0.5	Lee [17]
		2010	Nigeria	271	3	1.1	Sowemimo [14]
	Domestic pigs	2022	Ghana	56	1	1.8	Addy [16]
		1993–1994	Republic of Korea	662	9	1.4	Yang [18]
		2009–2010	Nigeria	226	11	4.9	Dogo [15]
	Subtotal			3765	37	1.0	
*Fecal egg counts*							
	Wild boars	2017	Nepal	100	44	44.0	Subedi [9]
	Subtotal			100	44	44.0	
*Urinalysis*							
	Domestic pigs	2016	Ecuador	364	84	23.0	Paccha [47]
	Subtotal			364	84	23.0	
Total				12,527	3037	24.2	

Necropsy/abattoir survey indicates adult worms recovered; fecal egg counts indicate egg detection in feces; urinalysis indicates egg detection in urine

*N/A* not available

### Wild boars as readily accessible hosts for *Stephanurus dentatus*

The lower prevalence in domestic pigs reflects controlled husbandry practices and anthelmintic use, whereas wild boars are more exposed to environmental infection via earthworms, which serve as paratenic hosts. *Stephanurus dentatus* has a direct life cycle: eggs are excreted in urine, L3 larvae can penetrate the skin or be ingested via earthworms which serve as paratenic hosts, and larvae migrate to mesenteric lymph nodes, the liver, and finally the perirenal region and ureters within 107–113 days. In modern indoor pig production, access to earthworms is minimal, limiting infection. In contrast, wild boars’ natural exposure to soil and earthworms makes them highly susceptible to infection [37, 38].

### Pathogenesis and clinical signs

Data on the pathogenetic effects of *S. dentatus* infection in wild boars were not available in the present study, as overall health status of the animals was not collected. However, *S. dentatus* infection in domestic pigs is known to cause substantial adverse effects on growth performance. Experimental studies demonstrated that final body weight and average daily gain declined significantly with increasing infection intensity; for example, uninfected pigs exhibited an average daily gain 69% greater than pigs administered 842 infective larvae per kilogram of body weight [39]. Similarly, Sapong (1972) reported that pigs orally administered approximately 2500 infective larvae on two occasions developed signs of emaciation and poor health approximately 6 months later [12]. In addition to growth retardation, *S. dentatus* infection may lead to eosinophilia, hepatic and pancreatic abscesses, extensive fibrosis [34], and, in rare cases, parasitic encephalitis, as reported in a pig in Brazil that exhibited left lateral recumbency, limb spasticity, marked prostration, and strabismus [40].

In the early stages of infection, percutaneous transmission of *S. dentatus* may result in subcutaneous nodules, lymph node enlargement, and stiffness of the limbs. As the infection progresses, affected pigs may exhibit reduced growth rate, anorexia, and emaciation. Additional pathological manifestations include eosinophilia, hepatic and pancreatic abscesses, and extensive fibrosis, primarily associated with larval migration [34, 37]. Clinical signs of *Stephanurus* infection are generally uncommon; however, moderate-to-heavy infections can reduce weight gain and production efficiency, occasionally causing atypical manifestations, such as thrombus formation in portal veins, hepatic artery, or caudal vena cava, and abscesses in lungs, pancreas, and lymph nodes [41].

### Identification keys

**Fig. 4:**
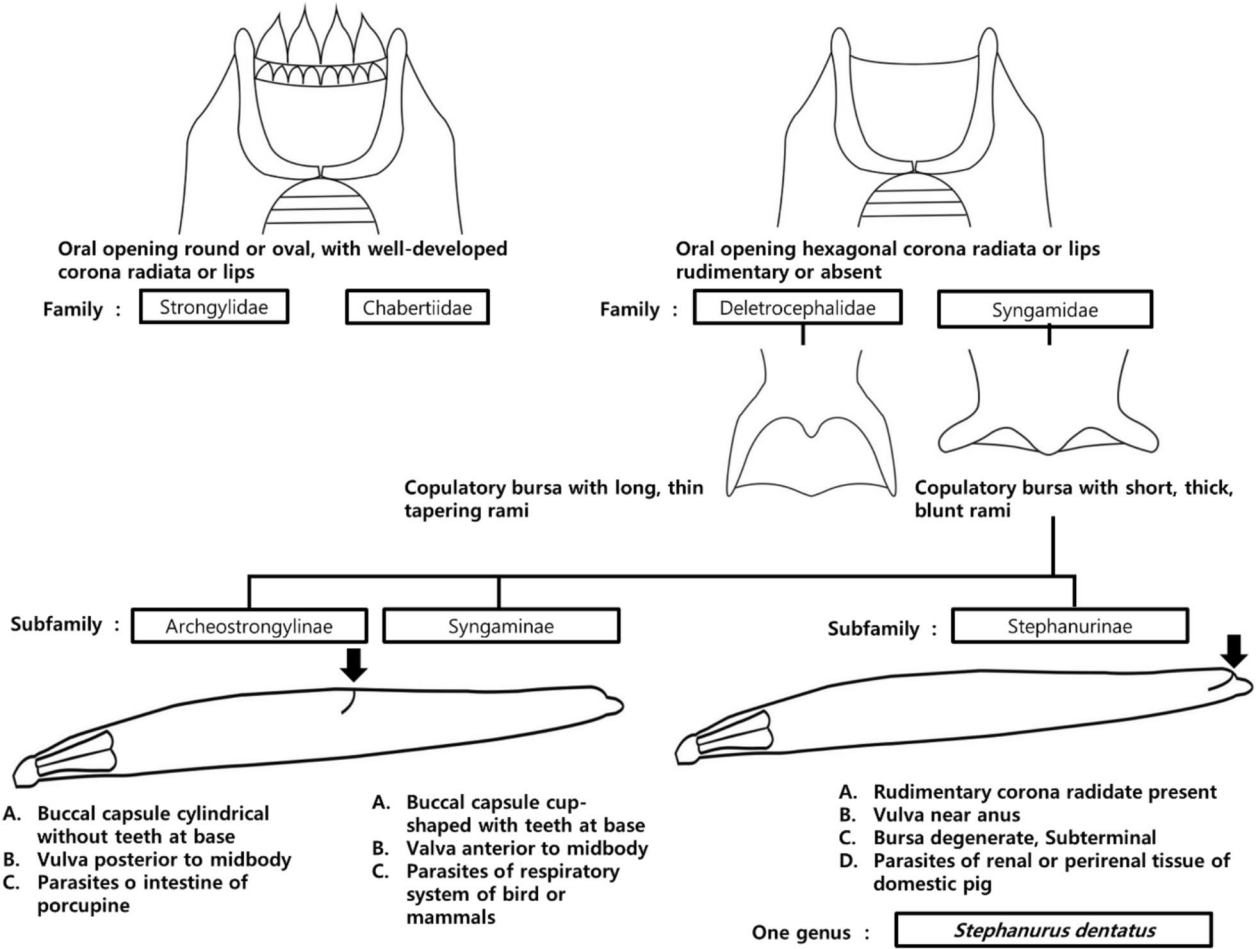
Taxonomic placement of *Stephanurus dentatus* (redrawn from Anderson, 2009 and Daubney 1923). Classification under the family Syngamidae

Identification keys based on the morphological characteristics of the family Syngamidae, subfamily Stephanurinae, and the genus *Stephanurus* are provided in this study (Fig. 4). The identification criteria were adapted from Anderson [42] and Daubney [25]. Morphological identification of *S. dentatus* is relatively straightforward because the genus *Stephanurus* contains a single species.

Species identification was based on several diagnostic features, including a rudimentary corona radiata, a vulva located near the anus, and a short, rudimentary male copulatory bursa (Fig. 1). According to Daubney [25], the distance from the anterior end to the excretory pore ranges from 0.5 to 0.6 mm, which differs slightly from the measurements obtained in the present study (0.94–1.05 mm). However, measurements of other morphological characters showed no significant discrepancies, and the overall morphological features were consistent with previous descriptions (Table 3).

Members of the family Syngamidae include parasites of the respiratory system of birds and mammals, the urinary system of swine, and the intestinal tract of Hyrax species. This family is characterized by the presence of a dorsal gutter, the absence of a perioral groove, a reduced copulatory bursa with short, thick spicules, and a vulva typically located at the midbody, with the exception of the genus *Stephanurus* [24].

### Reclassified taxonomy based on molecular analysis

Recent phylogenetic studies using concatenated amino acid sequences from 12 protein-coding genes support the hypothesis that *Stephanurus dentatus* is more closely related to members of the family Chabertiidae than to the family Syngamidae, in which it is currently classified [21, 22]. Concordantly, phylogenetic analysis of sequences generated in the present study also places *S. dentatus* within the family Chabertiidae (Fig. 3).

Within the family Syngamidae, the subfamilies Syngaminae and Archeostrongylinae are characterized by a vulva located near the midbody, whereas in the subfamily Stephanurinae, females of the genus *Stephanurus* have a vulva located near the anus (Fig. 1B, F) [20]. In addition, nematodes of the family Chabertiidae possess a type II ovejector, whereas those of the family Syngamidae typically have a type I ovejector, with the exception of the subfamily Stephanurinae, which also exhibits a type II ovejector [20, 25].

**Fig. 5:**
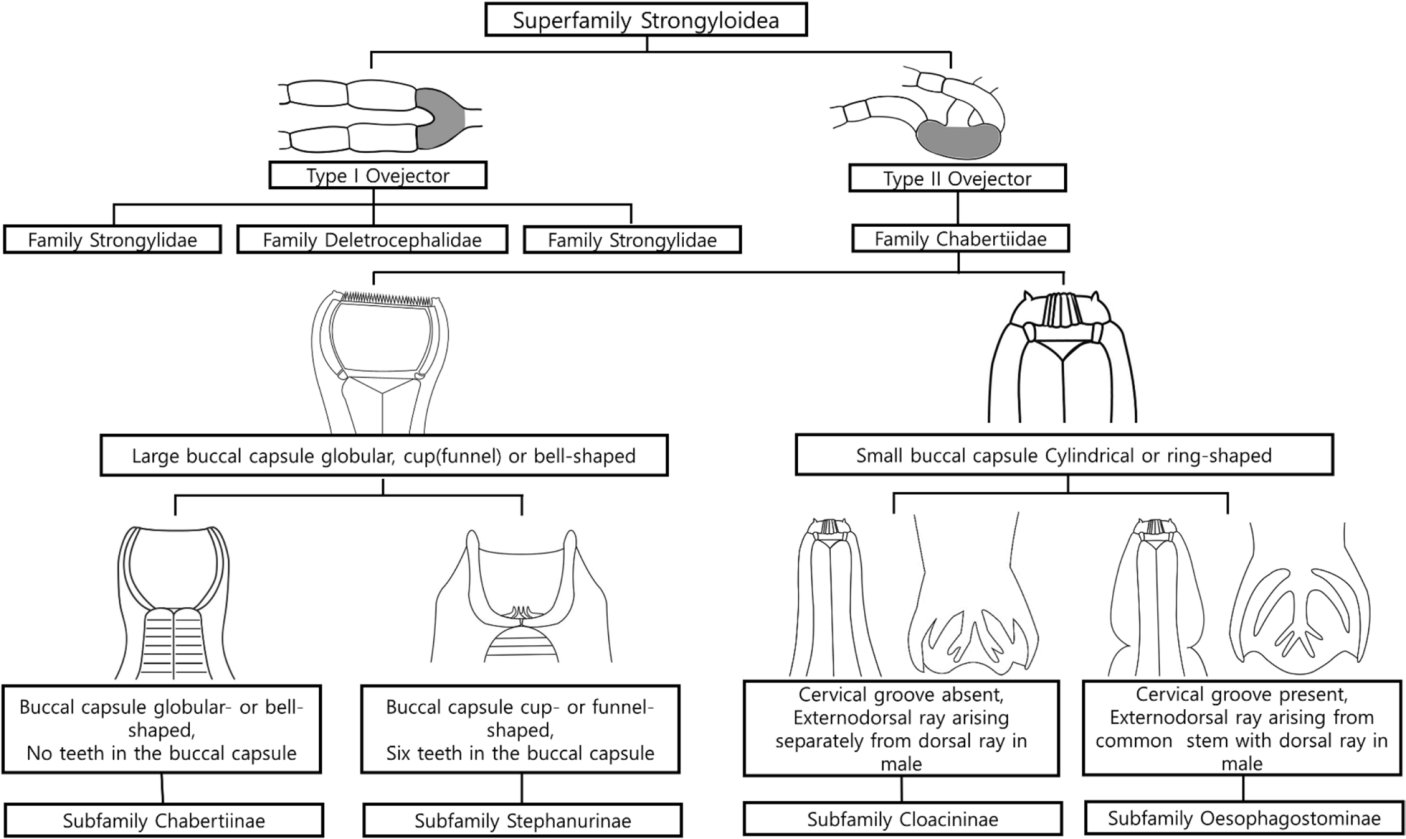
Taxonomic placement of *Stephanurus dentatus* (redrawn from Anderson, 2009 and Daubney 1923). Classification under the family Chabertiidae

Taken together, the molecular phylogenetic evidence and these morphological characteristics support the reclassification of the subfamily Stephanurinae from the family Syngamidae to the family Chabertiidae. Although *S. dentatus* remains the only known nematode species inhabiting the perirenal fat, ureters, and kidneys of its hosts, we provide revised identification keys for the subfamily Stephanurinae, newly placed within the family Chabertiidae, on the basis of morphological features (Fig. 5).

## Conclusions

This study provides the first epidemiological data on *Stephanurus dentatus* infection in wild boars inhabiting the southwestern regions of South Korea. The findings presented here, together with the detailed morphological descriptions and identification keys, will serve as valuable resources for faunistic and taxonomic studies of this parasite in pigs.

## Supplementary Information

**Additional file 1: Figure S1**. Geographic distribution of wild boars sampled in four southwestern regions of South Korea (n = 167). A Boseong-gun, B Suncheon-si, C Gwangyang-si, D Haman-si. Each dot indicates one animal. (PNG 1968 kb)

## Data Availability

Data supporting the main conclusions of this study are included in the manuscript.

## Abbreviations

OsO_4_: Osmium tetroxide
L1, L3: First and third stage larvae
