# Supplementary figures and images for: Epidemiology, morphology, and molecular characterization of Stephanurus dentatus (Nematoda: Syngamidae) in wild boars from southwestern South Korea
Source: Parasit Vectors. 2026 Apr 10;19:218. doi: 10.1186/s13071-026-07303-6 (PMC13185372; doi:10.1186/s13071-026-07303-6)

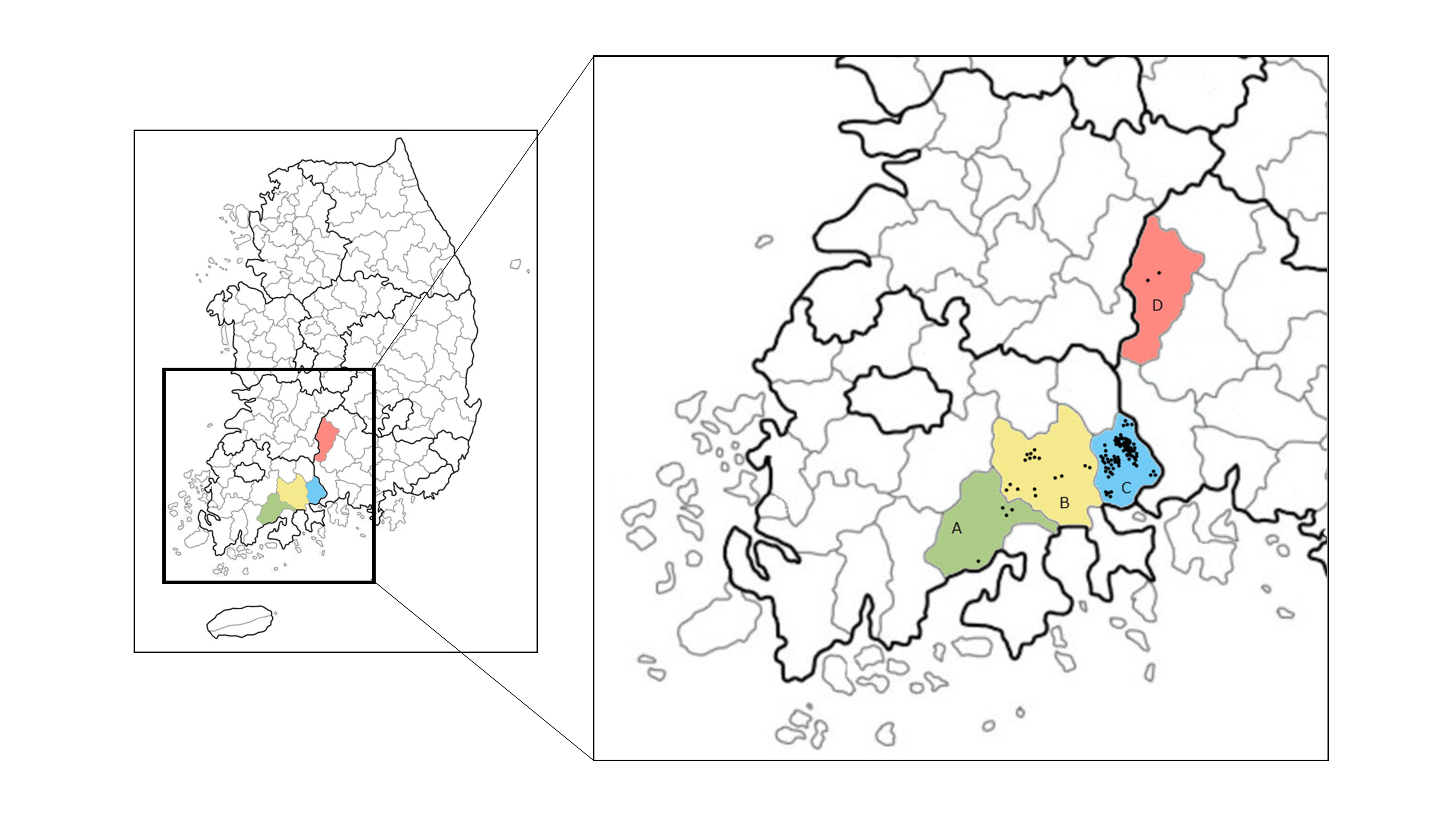

Supplement: Supplementary file 1 — Additional file 1: Figure S1. Geographic distribution of wild boars sampled in four southwestern regions of South Korea (n = 167). A Boseong-gun, B Suncheon-si, C Gwangyang-si, D Haman-si. Each dot indicates one animal. (PNG 1968 kb) [file 13071_2026_7303_MOESM1_ESM.png]
